# Effects of inspiratory muscle training on cardiorespiratory network physiology: evidence from cardiac autonomic modulation, respiratory sinus arrhythmia, and baroreflex sensitivity analysis

**DOI:** 10.3389/fnetp.2026.1761610

**Published:** 2026-02-17

**Authors:** Thiago Rodrigues Gonçalves, Selena Cristina Henriques Fontes, Michele Vaz Canena, Deysiane Peres da Silva Clemente de Oliveira, Pedro Paulo da Silva Soares, Gabriel Dias Rodrigues

**Affiliations:** Department of Physiology and Pharmacology, Fluminense Federal University, Niterói, Brazil

**Keywords:** cardiac autonomic control, cardiopulmonary interaction, cardiovascular control, respiratory control, respiratory muscle training

## Abstract

**Introduction:**

Inspiratory muscle training (IMT) has been proposed as a non-pharmacological strategy capable of improving respiratory performance and modulating cardiovascular autonomic function. However, its effects on baroreflex sensitivity, heart rate variability, and cardiorespiratory interactions in healthy young adults remain insufficiently understood. Therefore, this study aimed to determine whether a 4-week IMT program, performed at moderate load, improves inspiratory muscle strength, cardiac autonomic modulation, spontaneous baroreflex sensitivity (BRS), and respiratory pattern in healthy individuals.

**Methods:**

Twenty-two healthy young men were randomly assigned to an experimental group (60% of maximal inspiratory pressure, MIP) or a placebo group (10% of MIP). Before and after the intervention, participants underwent pulmonary function testing and assessments of inspiratory muscle performance, as well as hemodynamic, autonomic, and respiratory recordings during spontaneous and controlled breathing. Heart rate variability (HRV), blood pressure variability, and BRS (α-LF) were assessed during respiratory sinus arrhythmia (RSA), and responses to the Valsalva maneuver were also evaluated.

**Results:**

IMT significantly increased MIP by approximately 26% and enhanced peak inspiratory flow, without changes in pulmonary volumes. Vagal indices of HRV increased after training (rMSSD and HF; p ≤ 0.05), indicating enhanced parasympathetic modulation. IMT also modified the respiratory pattern, reducing the Ti/Ttot ratio and increasing expiratory time (p = 0.04). No significant changes were observed in blood pressure variability or BRS. RSA analysis demonstrated a reduction in inspiratory heart rate, and the Valsalva maneuver revealed attenuation of heart rate overshoot in phase IV.

**Discussion:**

In conclusion, a 4-week IMT program in healthy young adults improves inspiratory muscle performance, enhances vagally mediated HRV, and promotes adjustments in respiratory pattern, without altering spontaneous baroreflex sensitivity. These findings suggest that the autonomic benefits of IMT on cardiac vagal modulation are predominantly mediated by respiratory mechanisms.

## Introduction

The autonomic nervous system (ANS) is the primary effector of neural control over visceral function, regulating cardiovascular, respiratory, and other homeostatic processes through complex feedback loops. A key measure of this peripheral regulation is spontaneous baroreflex sensitivity (BRS), which quantifies the dynamic interplay between blood pressure and heart rate fluctuations (Karim et al., 2023; Fisher et al., 2022). These oscillations also manifest as cardiorespiratory coupling, a functional mechanism essential for optimizing physiological efficiency and adaptive responses. However, this peripheral autonomic function is not isolated; it is governed by a central-autonomic network, the core of the ‘mind-body’ connection. This network integrates key brain regions, including the insula, anterior cingulate, and prefrontal cortex, with brainstem cardiovascular centers to orchestrate adaptive responses to physical and psychological stressors (De et al., 2024; Rodrigues et al., 2018).

Inspiratory muscle training (IMT) involves training the diaphragm and accessory muscles. IMT is a viable alternative for increasing inspiratory muscle strength and physical respiratory capacity in healthy adults. It also influences cardiovascular and cerebrovascular control at rest and during stress conditions, such as physical exercise and orthostatic stress, in healthy elderly women (Rodrigues et al., 2018; Rodrigues et al., 2020a; Rodrigues et al., 2021a). In the elderly population, IMT improves cardiac autonomic control at rest and after exercise, cerebrovascular response to orthostatic stress, static and dynamic balance, blood pressure control, endothelial function, and oxidative stress (De et al., 2024; Rodrigues et al., 2020a). IMT also improved vagus nerve mediated heart rate variability (HRV) at rest during spontaneous breathing, as well as post-exercise vagal reactivation and chronotropic response during respiratory sinus arrhythmia (RSA) (Rodrigues et al., 2018). Recently, IMT has been shown to improve spontaneous breathing patterns and cardiac autonomic control, but not spontaneous baroreflex sensitivity (Rodrigues et al., 2021a).

Spontaneous breathing and RSA patterns appear to be possible mechanisms underlying improvements in cardiac vagal tone after IMT (Rodrigues et al., 2018; Rodrigues et al., 2021a). The RSA is a primary reflex phenomenon that receives information from baroreceptors (Karemaker, 2009). Due to the modulating effect of respiratory rhythm on heart rate, called cardiorespiratory coupling, it can influence cardiac and respiratory dynamics (Fisher et al., 2022). The shortening of the R-R interval during inspiration (increased heart rate) and its lengthening during expiration (decreased heart rate) reflect, respectively, the withdrawal and reactivation of cardiac vagal modulation, constituting a robust index of vagal function and respiratory–circulatory interactions. RSA improved HRV, particularly in the high-frequency band, reflecting parasympathetic activity, and baroreflex sensitivity in healthy adults during interventions combining HRV biofeedback and slow breathing (Giorgi and Tedeschi, 2025). The effects induced by IMT on RSA, breathing patterns, and baroreflex control interactions remain unclear in young, healthy individuals. The IMT has been shown to confer physiological benefits in healthy adults and to improve physical capacity (Ramsook et al., 2017).

In the current study, we aimed to test the hypothesis that inspiratory muscle training improves spontaneous baroreflex, HRV, and spontaneous breathing patterns in healthy individuals. Assessing inspiratory muscle performance and the interaction between respiratory and circulatory patterns is important across a range of contexts in pulmonary and cardiac health. Thus, this study aimed to analyze the impact of increased inspiratory muscle strength after IMT on cardiovascular autonomic modulation and IMT induced interaction between the respiratory and cardiovascular systems in healthy individuals.

## Methods

### Participants

A total of 22 young, healthy men (27 ± 4 years; 78.0 ± 12.1 kg; 178.5 ± 5.3 cm, and body mass index: 21.8 ± 3.4 kg/m^2^) participated in the present study. They were divided into 2 groups: the Experimental group (EXP) and the Placebo group (PLA). All participants were assessed using a 12-lead ECG (Wincardio™, Brazil), and a pulmonary function test was performed (Spirometer, Datalink, France). The selected volunteers did not present abnormal cardiac electrical function or pulmonary function outside normal limits (American Thoracic Society, 2002). The experimental protocol was approved by the Research Ethics Committee of the Faculty of Medicine of the Federal Fluminense University (UFF-Brazil) under number 39148514.9.0000.5243, and free and informed consent was obtained from all participants before the start of the study, through the signing of the consent form. Participants were instructed not to consume foods and beverages containing caffeine and to refrain from strenuous physical exercise (>5 metabolic equivalents) on the days of assessment.

### Experimental protocol

The experimental protocol consisted of two laboratory visits, one before the start of the inspiratory muscle training (IMT) protocol and another at the end of the 4-week IMT protocol. Volunteers were randomly allocated, by simple randomization, into two groups in a single-blind design (participants were unaware of the magnitude of the load): IMT performed with an effective load (EXP) or IMT performed with an ineffective load (PLA). It is important to note that the smaller final sample size in the PLA group resulted from the exclusion of data due to signal loss and/or discontinuation during the 4-week protocol. Hemodynamic, autonomic, and respiratory variables were recorded during both visits. Initially, all volunteers were acclimatized to the laboratory and underwent a 30-min familiarization process with the equipment and tests. During the IMT protocol, patients from both groups visited the laboratory three times to readjust the specific load. Before and after IMT, volunteers underwent pulmonary and inspiratory tests, as well as a resting assessment with spontaneous breathing in a seated position for 10 min. During the resting assessment, cardiovascular, autonomic, hemodynamic, and respiratory responses were analyzed. Tests were performed at 10-min intervals. In all assessments, blood pressure was recorded using infrared photoplethysmography (Finapress, Finometer, FMS, Netherlands) (Parati et al., 1989), heart rate by device-specific electrocardiogram (EKG module, Finapress, Finometer, FMS, Netherlands), and breath rate (BR) was assessed using a breathing belt (AD Instruments, United States). Data were monitored and synchronized using specific software (LabChart 8.0, AD Instruments, United States), with a sampling rate of 1,000 Hz. For autonomic responses, HRV, blood pressure variability (BPV), and BRS were investigated.

### Pulmonary and inspiratory tests

Three forced expiration maneuvers were performed at 3-min intervals to measure the pulmonary volumes and capacities, such as forced vital capacity (FVC), forced expiratory volume in 1 s (FEV1), and the peak expiratory flow (PEF). The best-validated expiratory maneuver with the highest value was used for analysis.

The maximal inspiratory pressure (MIP) was determined using a manovacuometer (GlobalMed, Brazil). For the determination of the MIP and maximal expiratory pressure (MEP), 10 consecutive inspiratory and expiratory maneuvers were performed. The highest value was selected for each. This was followed by 10 maximal inspirations with minimum load (3 cmH_2_O, the lower limit of the device) through an inspiratory resistor (K5 Powerbreathe, England) to determine the inspiratory flow peak (IFP) and the maximal inspiratory volume (MIV).

### Cardiorespiratory assessment

Participants remained seated for 10 min, breathing spontaneously. For hemodynamic assessment, blood pressure (BP) was recorded continuously and non-invasively using photoplethysmography (Finapress, Finometer, FMS, Netherlands). The last 5 min of each stage were used for analysis. Metabolic variables were measured using a metabolic gas analyzer (Ultima, MedGraphics, United States) and a medium flow pneumotachograph. The device was calibrated at the beginning of each test with a 3 L cylinder. Measurements of pulmonary ventilation (VE), tidal volume (Vt), BR, inspiratory time to total respiratory time ratio (Ti/Ttot), and expiratory time (Te) were performed.

### Heart rate and blood pressure variabilities

For HRV analysis, 5-min RR interval time series were analyzed using an automatic algorithm for artifact removal and manual inspection for ectopic beats and incorrect detections, and the beat-by-beat RR interval series were then converted into equally spaced time series with 200 m intervals using cubic spline interpolation. Spectral analysis was processed using Fast Fourier Transform (FFT) with the Welch method and a Hanning window with 50% overlap using a custom algorithm (Matlab 6.0, Mathworks Inc., United States) (Gonçalves et al., 2014).

Time domain analysis consisted of measures of RR intervals (average of all normal RR intervals), standard deviation of all normal RR intervals (SDNN), and the square root of the sum of successive differences between adjacent normal RR intervals squared (RMSSD).

In the frequency domain, the power spectrum density function was integrated in the three classic frequency bands, as follows: 1) Very low frequency band (VLF: 0.01–0.04 Hz); 2) Low frequency band (LF: 0.04–0.15 Hz); and 3) High frequency band (HF: 0.15–0.40 Hz). The HF was used as an index of vagal modulation. The LF component is influenced by both sympathetic and parasympathetic systems (Montano et al., 1994). The spectral values were expressed as normalized units (nu), which were calculated by dividing the power of each component by total power (TP), from which the VLF component had been subtracted, and then multiplying this value by 100 (LFnu and HFnu for normalized low and high frequency powers, respectively). The LF/HF ratio was adopted as a marker of sympathovagal balance.

The BPV was acquired by spectral analysis and described by the variance of the systolic blood pressure (var-SBP) and by the low frequency component of the SBP (LF-SBP). The BRS was evaluated by the alpha index for low (α-LF)-frequency components through the SBP and pulse interval recorded in Matlab language (Matlab 6.0, Mathworks Inc., United States).

The relationship between RR interval and systolic arterial pressure (SAP) was assessed to evaluate baroreflex function and cardiovascular complexity. Cross-spectral analysis was performed to calculate the squared coherence (k^2^) in the low-frequency band between RR and SAP for each participant. Coherence values range from 0% (no linear correlation) to 100% (perfect linear correlation), with a threshold of >50% indicating significant coupling between the signals. The phase (Ph) and gain were also derived from this analysis to determine the direction and strength of the RR–SAP relationship; an increased gain and a negative phase within the low-frequency band are indicative of improved baroreflex control (De Abreu et al., 2020; Cairo et al., 2021). Cardiovascular complexity was evaluated using entropy-derived measures (De Abreu et al., 2020; Cairo et al., 2021). Corrected conditional entropy (CE) quantifies the information content and predictability of RR and SAP time series, ranging from 0 (future values completely predictable) to 1 (future values completely unpredictable). The index of regularity (Ro) is derived from CE and ranges from 0 (lowest regularity, highest complexity) to 1 (highest regularity, lowest complexity). Physiologically, increased regularity (higher Ro) and/or decreased entropy (lower CE) reflect a shift toward greater sympathetic modulation of the cardiovascular system (De Abreu et al., 2020; Cairo et al., 2021). All analyses were performed using Heart Scope II software (Amps LLC, New York, United States).

### Cardiovascular autonomic tests

Autonomic tests selected were the RSA and Valsalva maneuver. The RSA was performed in a sitting position with controlled breathing (6 rpm) by audiovisual metronome for 5 min, with recording of the hemodynamic variables recorded by FMS. The RSA dynamic was analyzed during the last minute of the test, obtaining averages of the HR values during inspiratory and expiratory cycles. Furthermore, the difference between HR inspiratory peak and HR expiratory decline, and the ratio between HR inspiratory peak and HR expiratory decline were used for RSA analysis. The Valsalva maneuver (Baker et al., 2024; Srivastav et al., 2025) was performed in a sitting position through a mercury column with 15 s duration. Before the maneuver, the participant took a deep inspiration and then immediately performed a sustained expiratory effort against a pressure of 40 mmHg for 15 s (phase II). After the effort, the participant remained seated while the hemodynamic responses characteristic of phases III and IV, including the overshoot in phase IV, were recorded.

### Inspiratory muscle training

IMT was performed using an inspiratory resistor portable device (Powerbreathe, United Kingdom) for 30 days with a single load of 60% MIP for the EXP group, and 10% MIP for the PLA group, acquired by manovacuometry test. Each volunteer was asked to perform 30 breaths per inspiratory exercise twice a day, every day. Weekly, individuals attended the laboratory for adjustment of 60% MIP or 10% MIP workload, as increased inspiratory muscle strength was seen during the training (Rodrigues et al., 2021b; Witt et al., 2007; Chiappa et al., 2008).

### Statistical analysis

The sample size was calculated to ensure statistical power greater than 0.80. Normality was assessed using the Shapiro-Wilk test, and data are presented as mean ± standard deviation. The Student’s t-test for independent samples was used to compare anthropometric characteristics and inspiratory variables between the EXP and PLA groups at baseline. A two-way repeated measures ANOVA was applied to assess pre- and post-IMT differences, followed by a Bonferroni *post hoc* test for multiple comparisons. The significance level was set at α ≤ 0.05, and all analyses were performed using SPSS software, version 20.0 (IBM, United States).

## Results

The study included fifteen healthy individuals allocated to the experimental group (26 ± 4 years; 77.6 ± 12.0 kg; 177.4 ± 5.5 cm; BMI 21.9 ± 3.3 kg/m^2^) and seven healthy individuals in the placebo group (28 ± 5 years; 78.6 ± 13.2 kg; 180.9 ± 4.3 cm; BMI 21.8 ± 3.9 kg/m^2^). The anthropometric and demographic characteristics of the participants are presented in Table 1.

**TABLE 1:** Demographic characteristics of the participants.

Characteristics	PLA	EXP
Age (years)	28 ± 5	26 ± 4
Body mass (kg)	78.6 ± 13.2	77.6 ± 12.0
Height (cm)	180.9 ± 4.3	177.4 ± 5.5
BMI (kg/m^2^)	21.8 ± 3.9	21.9 ± 3.3

PLA, placebo group; EXP, experimental group; BMI, body mass index.

**FIGURE 1:**
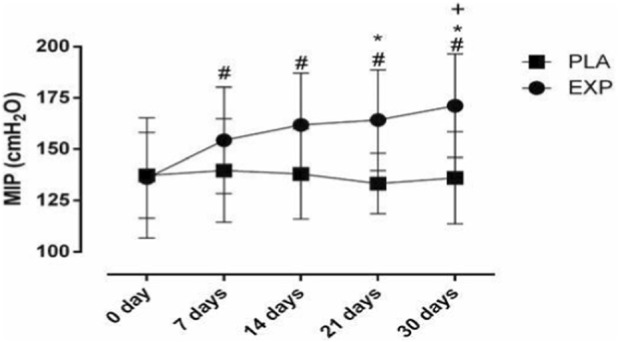
Changes in the weekly MIP during IMT. #, significant difference at baseline; *, significant difference at day 7, +, significant difference at day 14. MIP, maximal inspiratory pressure; PLA, placebo group; EXP, experimental.

After IMT, a significant increase in MIP was observed in the experimental group (pre: 135.2 ± 15.1; post: 170.5 ± 21.5 cmH_2_O; p < 0.001), while the placebo group showed no changes. This result is illustrated in Figure 1.

The other respiratory variables, FVC, FEV_1_, PEF, MEP, and IV, remained unchanged in both groups after IMT (p > 0.05). However, there was an increase in IPF (pre: 8.0 ± 1.1; post: 8.9 ± 1.0 L/s; p = 0.02). These results are shown in Table 2.

**TABLE 2:** Pulmonary and inspiratory tests before and after IMT.

Variable	PLA		EXP		​
	PRE_IMT	POST_IMT	PRE_IMT	POST_IMT	p-value
FVC (l)	4.9 ± 0.5	4.8 ± 0.4	4.9 ± 0.5	4.9 ± 0.5	0.45
FEV_1s_ (l)	4.2 ± 0.6	3.7 ± 0.5	4.0 ± 0.6	4.0 ± 0.6	0.14
PEF (l/s)	8.8 ± 1.8	8.4 ± 1.7	8.9 ± 1.8	9.3 ± 1.2	0.40
MEP (cmH_2_O)	116.0 ± 29.0	123.3 ± 27.6	120.3 ± 23.3	121.4 ± 20.9	0.42
MIP (cmH_2_O)	137.3 ± 20.1	136.1 ± 22.4	135.2 ± 15.1	170.5 ± 21.5^#^	0.00
IV (l)	3.4 ± 0.4	3.5 ± 0.5	3.4 ± 0.4	3.5 ± 0.5	0.23
IPF (l/s)	8.4 ± 1.6	8.4 ± 1.5	8.0 ± 1.1	8.9 ± 1.0^#^	0.02

IMT, inspiratory muscle training; FVC, forced vital capacity; FEV1s, forced expiratory volume in one second; EPF, expiratory peak flow; MEP, maximal expiratory pressure; MIP, maximal inspiratory pressure; IV, inspiratory volume; IPF, inspiratory peak flow; IMT, inspiratory muscle training; PLA, placebo group; EXP, experimental group; #: significant difference.

Hemodynamic variables did not change after IMT. During spontaneous breathing, no significant differences were observed in systolic blood pressure (111.5 ± 16.5 vs. 108.7 ± 13.1 mmHg; p = 0.79), diastolic blood pressure (57.5 ± 6.8 vs. 57.5 ± 6.2 mmHg; p = 0.92), or mean blood pressure (75.5 ± 8.6 vs. 74.5 ± 7.6 mmHg; p = 0.55) in the experimental group. Blood pressure variability (SBP-var: 61.9 ± 22.0 vs. 61.5 ± 26.6 ms^2^/mmHg; p = 0.25), the low-frequency component of systolic pressure variability (SBP-LF: 17.0 ± 5.2 vs. 17.4 ± 8.4 ms^2^/mmHg; p = 0.09), and baroreflex sensitivity (αLF: 11.7 ± 4.9 vs. 12.9 ± 4.5 ms/mmHg; p = 0.46) also remained unchanged in both groups. During controlled breathing, values were similarly stable: mean arterial pressure (72.5 ± 8.3 vs. 72.8 ± 7.7 mmHg; p = 0.76) and αLF (9.9 ± 2.9 vs. 9.7 ± 3.3 ms/mmHg; p = 0.96), as shown in Table 3.

**TABLE 3:** BPV, BRS, and RSA at rest before and after IMT.

Variable	PLA		EXP		​
	PRE_IMT	POST_IMT	PRE_IMT	POST_IMT	p-value
Spontaneous breathing					
SBP (mmHg)	114.8 ± 21.3	114.0 ± 12.3	111.5 ± 16.5	108.7 ± 13.1	0.79
DBP (mmHg)	61.3 ± 14.5	60.7 ± 11.9	57.5 ± 6.8	57.5 ± 6.2	0.92
MBP (mmHg)	79.1 ± 16.1	74.7 ± 11.0	75.5 ± 8.6	74.5 ± 7.6	0.55
SBP-var(ms^2^/mmHg)	52.0 ± 17.0	40.0 ± 17.6	61.9 ± 22.0	61.5 ± 26.6	0.25
SBP-LF (ms^2^/mmHg)	9.7 ± 9.9	18.6 ± 12.8	17.0 ± 5.2	17.4 ± 8.4	0.09
αLF (ms/mmHg)	9.7 ± 2.4	12.6 ± 5.0	11.7 ± 4.9	12.9 ± 4.5	0.46
Controlled breathing					
SBP (mmHg)	114.4 ± 22.3	110.8 ± 15.7	107.0 ± 14.5	106.6 ± 12.6	0.66
DBP (mmHg)	59.1 ± 15.2	59.5 ± 14.0	55.3 ± 6.2	55.9 ± 6.9	0.85
MBP (mmHg)	78.1 ± 17.2	76.6 ± 14.3	72.5 ± 8.3	72.8 ± 7.7	0.76
SBP-var (ms^2^/mmHg)	44.2 ± 25.8	37.0 ± 23.8	55.8 ± 19.3	67.9 ± 42.9	0.28
SBP-LF (ms^2^/mmHg)	11.7 ± 5.3	10.7 ± 9.8	16.7 ± 9.1	14.9 ± 8.3	0.89
αLF (ms/mmHg)	9.8 ± 2.8	9.7 ± 2.3	9.9 ± 2.9	9.7 ± 3.3	0.96
RSA					
HR_INS_ (bpm)	86.6 ± 7.0	86.4 ± 6.4	92.3 ± 17.4	84.9 ± 14.5	0.01^#^
HR_EXP_ (bpm)	59.2 ± 5.7	59.6 ± 3.5	63.3 ± 12.4	60.9 ± 9.1	0.46
ΔHR_INS-EXP_ (bpm)	27.4 ± 5.4	25.9 ± 7.4	28.9 ± 10.6	24.1 ± 8.3	0.10
HR_INS/EXP_ (bpm)	1.5 ± 0.1	1.4 ± 0.1	1.5 ± 0.2	1.4 ± 0.1	0.20

IMT, inspiratory muscle training; BPV, blood pressure variability; RSA, respiratory sinus arrhythmia; SBP, systolic blood pressure; DBP, Diastolic blood pressure SBP-var, variance of systolic blood pressure; SBP-LF, low-frequency component of systolic blood pressure; αLF, alpha index in the low-frequency band; HR, heart rate; Δ, delta; #, significant difference.

In contrast, cardiac autonomic modulation improved after IMT. During spontaneous breathing, rMSSD increased from 51.7 ± 27.8 to 69.1 ± 26.4 ms (p = 0.05), and HF rose from 1,278.2 ± 1,606.6 to 2,161.2 ± 1,611.3 ms^2^ (p = 0.03), indicating enhanced vagal modulation. Mean R-R interval increased from 847.4 ± 149.7 to 907.1 ± 142.1 ms (p = 0.13), although without statistical significance. During controlled breathing, rMSSD also increased from 46.6 ± 22.2 to 56.3 ± 28.5 ms (p = 0.05), while the remaining HRV indices showed no changes (p > 0.05), as presented in Table 4.

**TABLE 4:** HRV at rest before and after IMT.

Variable	PLA		EXP		​
	PRE_IMT	POST_IMT	PRE_IMT	POST_IMT	p-value
Spontaneous breathing					
R-R (ms)	880.7 ± 60.2	882.6 ± 69.8	847.4 ± 149.7	907.1 ± 142.1	0.13
SDNN (ms)	70.6 ± 16.9	68.9 ± 25.4	79.5 ± 29.6	92.1 ± 29.6	0.20
rMSSD (ms)	47.0 ± 17.0	50.5 ± 22.1	51.7 ± 27.8	69.1 ± 26.4^#^	0.05
LF (ms^2^)	1873.5 ± 1913.4	1590.5 ± 1846.8	2305.6 ± 1537.5	2750.4 ± 1543.2	0.48
HF (ms^2^)	922.6 ± 740.0	1134.3 ± 815.0	1,278.2 ± 2,606.6	1,161.2 ± 2,611.3^#^	0.03
LF (u.n)	38.7 ± 21.4	42.1 ± 20.7	43.3 ± 8.8	46.8 ± 24.0	1.00
HF (u.n)	22.8 ± 17.0	26.1 ± 22.1	22.5 ± 12.8	35.1 ± 22.7	0.33
LF/HF	3.1 ± 2.2	2.6 ± 1.7	2.7 ± 2.1	1.6 ± 1.0	0.49
Controlled breathing					
R-R (ms)	850.3 ± 67.8	850.7 ± 80.3	820.5 ± 164.5	856.3 ± 148.6	0.34
SDNN (ms)	58.6 ± 13.6	54.4 ± 15.6	67.3 ± 17.7	68.7 ± 23.5	0.55
rMSSD (ms)	44.8 ± 13.5	40.2 ± 16.6	46.6 ± 22.2	56.3 ± 28.5^#^	0.05
LF (ms^2^)	1,211.0 ± 729.1	855.3 ± 645.4	1,620.7 ± 1,016.0	1,467.8 ± 2,369.9	0.77
HF (ms^2^)	859.7 ± 520.1	833.3 ± 824.9	1,099.7 ± 1,124.1	1,577.0 ± 1,371.0	0.16
LF (nu)	41.8 ± 16.6	37.5 ± 25.3	44.5 ± 18.7	34.5 ± 15.1	0.55
HF (nu)	35.7 ± 21.7	33.5 ± 22.2	30.2 ± 18.4	35.7 ± 13.1	0.20
LF/HF	2.5 ± 3.4	1.8 ± 1.6	3.4 ± 5.0	1.3 ± 1.2	0.46

IMT, inspiratory muscle training; HRV, heart rate variability; R–R, mean of normal RR, intervals; SDNN, standard deviation of normal RR, intervals; rMSSD, square root of the mean of successive differences between adjacent normal RR, intervals squared; LF, low-frequency component; HF, high-frequency component; LF/HF, sympathovagal balance; #, significant difference.

Expiratory time increased after IMT in the experimental group (2.87 ± 0.52 vs. 3.57 ± 1.29 s; p = 0.05), with no modifications in the placebo group. Consistently, the Ti/Ttot ratio decreased in the experimental group (0.32 ± 0.04 vs. 0.29 ± 0.02 s; p = 0.04), as shown in Table 5. All other respiratory variables remained unchanged (p > 0.05).

**TABLE 5:** Ventilatory data at rest before and after IMT.

Variable	PLA		EXP		​
	PRE_IMT	POST_IMT	PRE_IMT	POST_IMT	p-value
Spontaneous breathing					
VE (L/min)	9.41 ± 1.18	10.91 ± 2.45	10.80 ± 0.90	9.98 ± 0.63	0.08
BR (bpm)	13.57 ± 3.87	14.08 ± 2.37	15.34 ± 3.23	13.70 ± 3.96	0.22
Ti/Ttot	0.30 ± 0.04	0.31 ± 0.03	0.32 ± 0,04	0.29 ± 0.02^#^	0.04
Te (s)	3.55 ± 1.18	3.15 ± 0.44	2.87 ± 0.52	3.57 ± 1.29^#^	0.05
V_t_ (mL)	749 ± 151	826 ± 287	747 ± 151	817 ± 151	0.94
Controlled breathing					
VE (L/min)	11.17 ± 2.10	11.68 ± 2.57	14.99 ± 3.39	16.67 ± 4.38	0.59
BR (bpm)	14.74 ± 0.59	15.14 ± 0.16	14.89 ± 0.25	14.80 ± 0.37	0.12
Ti/Ttot	0.32 ± 0.04	0.33 ± 0.03	0.35 ± 0.04	0.32 ± 0.03^#^	0.05
Te (s)	2.81 ± 0.22	2.78 ± 0.12	2.68 ± 0.24	2.84 ± 0.31^#^	0.01
V_t_ (mL)	761 ± 123	779 ± 173	1,019 ± 228	1,130 ± 278	0.51

IMT, inspiratory muscle training; VE, minute ventilation; BR, breathing rate; Te, expiratory time; Vt, tidal volume; PLA, placebo group; EXP, experimental group; #, significant difference.

In the RSA analysis, a significant reduction in inspiratory heart rate was observed, decreasing from 92.3 ± 17.4 to 84.9 ± 14.5 bpm (p = 0.01), whereas expiratory heart rate remained unchanged (63.3 ± 12.4 vs. 60.9 ± 9.1 bpm; p = 0.46). Similarly, the HRins/HRexp ratio did not differ after training (1.5 ± 0.2 vs. 1.4 ± 0.1; p = 0.20), as shown in Table 3.

**FIGURE 2:**
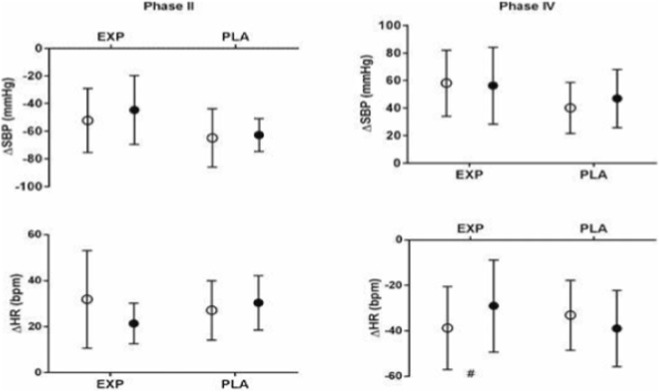
SBP and HR dynamics during phases II and IV of the Valsalva maneuver in the EXP and PLA groups. Open circles, pre IMT; filled circles, post IMT; PLA, placebo group; EXP, experimental group; ΔSBP, delta systolic blood pressure; ΔHR, delta Heart rate; #, significant difference.

During the Valsalva maneuver, the compensatory heart rate response in phase IV was attenuated after training (p < 0.05). The experimental group exhibited a smaller decrease in heart rate during phase IV compared with the placebo group, indicating a less pronounced parasympathetic overshoot following IMT. The systolic blood pressure responses in phases II and IV did not differ between groups (ΔSBP phase II: ≈ −50 to −60 mmHg; ΔSBP phase IV: ≈ +50 to +60 mmHg; p > 0.05). The chronotropic response in phase II also remained stable (ΔHR ≈ +20 bpm; p > 0.05). These findings, presented in Figure 2, suggest that IMT modulated the late vagal response without significantly altering the blood pressure component of the maneuver.

Advanced measures of cardiorespiratory coupling, including coherence, phase synchronization, gain, and transfer entropy, were also analyzed; however, no significant changes were observed following IMT in this cohort (Supplementary Material, Supplementary Table S1).

## Discussion

The main finding of this study was that 4 weeks of IMT increased inspiratory muscle strength and vagally mediated HRV at rest, without affecting BRS in healthy young men. In addition, IMT modified the spontaneous breathing cycle by shortening inspiratory time and prolonging expiratory time, thereby altering the pattern of respiratory sinus arrhythmia.

The 26% increase in Inspiratory muscle strength observed in the EXP group confirms the effectiveness of the protocol in improving inspiratory muscle strength, consistent with previous studies in healthy adults and older individuals (Rodrigues et al., 2018; Rodrigues et al., 2021b; Brown et al., 2014; DeLucia et al., 2017; Mills et al., 2014). As expected, the PLA group, trained at 10% of MIP, showed no significant changes (−0.9%), reinforcing that loads below this threshold do not elicit gains in inspiratory strength (Rodrigues et al., 2018; Rodrigues et al., 2021b). The additional increase in PIF following IMT suggests enhanced inspiratory muscle efficiency (Janssens et al., 2007; McConnell, 2013).

The training protocol was based on previous studies indicating that moderate-to-high inspiratory loads elicit greater gains in inspiratory muscle strength and are more consistently associated with autonomic adaptations (Rodrigues et al., 2018; Rodrigues et al., 2021b; Brown et al., 2014; DeLucia et al., 2017; Mills et al., 2014), whereas lower loads (≤30% MIP) show minimal effects (Cutrim et al., 2019; Vranish and Bailey, 2016). The 4-week duration was selected as the minimum period reported to induce neural and functional adaptations in inspiratory muscle performance (Rodrigues et al., 2018; Rodrigues et al., 2021b; Brown et al., 2014; DeLucia et al., 2017; Mills et al., 2014), while longer interventions may be required to promote vascular or baroreflex adaptations. Adherence to the IMT protocol exceeded 75%, comparable to previous home-based IMT studies, with weekly supervised sessions for load adjustment and technique verification and daily training logs used to monitor compliance (Rodrigues et al., 2021b; Ferreira et al., 2025).

Conversely, FVC, FEV1, and PEF remained unchanged, as previously reported in young (DeLucia et al., 2017) and older healthy individuals (Rodrigues et al., 2021b; Mills et al., 2014), as well as in clinical conditions such as diabetes mellitus (Kaminski et al., 2015), COPD (Cutrim et al., 2019), and obstructive sleep apnea (Vranish and Bailey, 2016; Ramos-Barrera et al., 2020).

The observed increases in rMSSD and HF at rest indicate enhanced cardiac vagal modulation, reflecting improved parasympathetic autonomic regulation. This is the first study to demonstrate such an effect in healthy young adults following a moderate-load IMT protocol. During the respiratory cycle, vagal control decreases during inspiration and is restored during expiration, allowing physiological oscillations in heart rate (Shao et al., 2024).

The breathing pattern, therefore, directly influences autonomic dynamics, larger tidal volumes and slower respiratory rates enhance vagal modulation and respiratory sinus arrhythmia (Rodrigues et al., 2018; Rodrigues et al., 2020b; Dick et al., 2014). Calabrese and collaborators (2000) demonstrated a positive correlation between HRV and respiratory period in healthy young adults, reinforcing that a longer expiratory time, as observed in the present study, favors vagal predominance (Rodrigues et al., 2018; Rodrigues et al., 2021b; Calabrese et al., 2000). Under resistive loading, the increase in negative intrathoracic pressure enhances the transmural gradient of the aorta and activates arterial baroreceptors, reflexively modulating cardiac autonomic control (DeLucia et al., 2021; De Abreu et al., 2017). Acute studies have confirmed this mechanism, showing increased vagal modulation during intermittent inspiratory efforts (Rodrigues et al., 2020b; De Abreu et al., 2017).

Changes in breathing pattern may also affect central levels of autonomic control through cardiorespiratory coupling (Rodrigues et al., 2021a; Dick et al., 2014). The nucleus tractus solitarius (NTS), by integrating chemoreceptive, mechanoreceptive, and baroreflex afferent inputs, may amplify and sustain vagal effects induced by larger tidal volumes and slower respiratory rhythms (Doehner et al., 2018; Sevoz-Couche and Laborde, 2022; Valenza et al., 2025). Neuroimaging evidence supports this central integration, showing that increases in tidal volume are associated with greater activation of dorsal medullary regions, including the NTS (Critchley et al., 2015). In addition, enhanced pulmonary vagal afferent input, together with modulation of limbic–cortical circuits involving the medial prefrontal cortex and the insula, resulting from alterations in breathing pattern, may promote a sustained reduction in sympathetic activity (Shao et al., 2024; Sevoz-Couche and Laborde, 2022; Ma et al., 2017; Jerath et al., 2015; Dampney, 2019).

Abreu and collaborators (2017) demonstrated that low-intensity IMT may chronically increase parasympathetic and/or reduce sympathetic cardiac modulation in patients with diabetes, hypertension, heart failure, and gastroesophageal reflux disease (De Abreu et al., 2017). Collectively, these findings support the concept that improved respiratory muscle efficiency achieved through IMT translates into enhanced cardiac autonomic control across diverse clinical populations (Rodrigues et al., 2018; Witt et al., 2007; Cutrim et al., 2019; Vranish and Bailey, 2016; Ramos-Barrera et al., 2020; Kaminski et al., 2015; Mello et al., 2012).

The magnitude of this autonomic response appears to depend on training load and duration. Protocols lasting ≥8 weeks with loads of 30% of MIP have been shown to improve autonomic control in adults with essential hypertension (Ferreira et al., 2011), COPD (Cutrim et al., 2019), and diabetes mellitus (Kaminski et al., 2015). Moderate-to-high loads (40%–75%) applied for up to 6 weeks have been effective in individuals with Parkinson’s disease (Ferreira et al., 2025), obstructive sleep apnea (Vranish and Bailey, 2016; Ramos-Barrera et al., 2020), and heart failure (Tanriverdi et al., 2023). These adaptations appear reversible, as reductions in inspiratory strength after a 6-week detraining period are accompanied by decreased autonomic modulation and regression of blood pressure improvements (Rodrigues et al., 2021a; Ramos-Barrera et al., 2020).

In the present study, IMT did not induce changes in BPV or BRS. Arterial baroreflex regulation depends on the pulsatile stretch of the carotid sinus and aortic arch walls, which activate mechanoreceptors that inhibit sympathetic tone and enhance vagal activity, resulting in reductions in heart rate and cardiac output (Ramos-Barrera et al., 2020; Parati et al., 2000). Although resistive inspiratory efforts generate oscillations in intrathoracic pressure and stroke volume that can transiently modulate arterial baroreceptor discharge, these effects do not translate into sustained autonomic adaptations (Witt et al., 2007; De Abreu et al., 2020).

This finding is consistent with studies conducted in healthy young and older adults, in which inspiratory muscle training (IMT) did not alter blood pressure or spontaneous baroreflex sensitivity, even after prolonged periods of training (Rodrigues et al., 2021a; DeLucia et al., 2017; De Abreu et al., 2017; De Abreu et al., 2020). Additional evidence indicates that, even when assessed using sensitive phase-based analytical methods, IMT interventions exert limited effects on central cardiorespiratory synchronization, reinforcing the absence of detectable baroreflex adaptations (Cairo et al., 2021). In contrast, guided slow breathing interventions without inspiratory load have demonstrated increases in baroreflex sensitivity in both healthy adults and patients with hypertension, suggesting that sustained reductions in respiratory rate may be the main determinant of these adaptations (Giorgi and Tedeschi, 2025; Krüerke et al., 2018).

Complementarily, clinically meaningful reductions in SBP associated with IMT appear to depend on longer interventions or higher inspiratory loads (Witt et al., 2007; Ferreira et al., 2011; Vranish and Bailey, 2016; De Abreu et al., 2017; Dampney, 2019). Although improvements in endothelial and vascular function as well as in inflammatory markers have been reported following IMT, these mechanisms may require longer exposure to translate into measurable hemodynamic changes (De Abreu et al., 2017; Ma et al., 2017; Dampney, 2019; Mello et al., 2012). In this context, despite the progressive increase in maximal inspiratory pressure observed over 4 weeks, the absence of concomitant changes in BPV reinforces the interpretation that the protocol duration was insufficient to induce detectable structural cardiovascular or baroreflex adaptations (Rodrigues et al., 2021a; Jerath et al., 2015; Tanriverdi et al., 2023).

In the present study, IMT demonstrated a significant impact on cardiac autonomic modulation, as evidenced by resting HRV analysis. However, we can observe a modification of the response to RSA and the Valsalva Maneuver, indicating that IMT reorganizes cardiorespiratory coupling and autonomic responsiveness to specific stress stimuli. In the RSA maneuver, we observed an attenuation in the increase of HR during inspiration (HRins) in the EXP group post-IMT.

This modification of the RSA response is intrinsically linked to the alteration in the controlled breathing pattern: the EXP group exhibited a shortening in Ti/Ttot and a consequent prolongation of the expiratory time (Te). While RSA is classically viewed as a marker of vagal modulation where reflex bradycardia is maximized during expiration, recent expert recommendations propose the term ‘Respiratory Heart Rate Variability (RespHRV) to emphasize that these oscillations are fundamentally driven by central coupling between the respiratory central pattern generator and cardiac vagal preganglionic neurons (Parati et al., 1989). Therefore, by prolonging Te and shortening the time when vagal input is inhibited, IMT likely optimizes this central neural integration. The extended expiratory phase provides a broader temporal window for the brainstem respiratory oscillator to drive rhythmic cardiac parasympathetic activity (Karim et al., 2023). This finding corroborates studies that associate the improvement in IMT-induced HRV with the reorganization of breathing patterns (Rodrigues et al., 2018; Rodrigues et al., 2021b; Mello et al., 2012).

From a physiological point of view, the strengthening of the inspiratory musculature, particularly the diaphragm, reduces respiratory work demand and the metabolic cost of breathing (Mello et al., 2012; Rodrigues and McConnell, 2024). This demand reduction may lead to less activation of Group III/IV muscle afferents and, consequently, an attenuation of the respiratory muscle metaboreflex. The reduced peripheral sympathetic stimulation stemming from fatigued or stressed respiratory muscles could, in turn, facilitate the observed increase in central vagal tone (Mello et al., 2012). The change in the breathing pattern with a greater Te induced by IMT is a key mechanism that, along with the improvement in muscle strength, suggests central modulation that optimizes cardiovagal timing in response to the respiratory cycle.

Additionally, IMT modified the hemodynamic response to the Valsalva Maneuver, a stress maneuver that assesses the integrity of the baroreflex arc and the capacity for sympathetic and vagal compensation. The attenuation of the HR response during Phase IV (overshoot) in the EXP group is an important finding. Although the spontaneous BRS Sensitivity at rest (evaluated by the alpha-LF index) was not significantly altered, the modification of the HR response to an acute stressor like Valsalva suggests that IMT is modulating the dynamics of the autonomic response, and not just the basal tone. This attenuation of vagal bradycardia in Phase IV may reflect a more balanced baroreflex system, capable of modulating HR more precisely after sympathetic discharge. Recent findings, such as the improvement of the autonomic response to orthostatic stress after IMT in patients with Parkinson’s Disease (Ferreira et al., 2025), reinforce the hypothesis that the intervention acts by improving dynamic autonomic responses.

The absence of significant change in advanced coupling measures (Supplementary Table S1) suggests that in our cohort, which lacked baseline autonomic dysfunction, IMT primarily improves cardiac vagal control and cardiorespiratory interactions (as reflected in spontaneous duty cycle and respiratory sinus arrhythmia response) rather than recoupling the fundamental network dynamics of cardiorespiratory interaction (phase synchronization, gain).

Although RSA, recently redefined as RespHRV by Menuet et al. (2025), is predominantly mediated by rhythmic parasympathetic activity, additional cardiorespiratory coupling mechanisms play fundamental roles outside this exclusively vagal pathway (Menuet et al., 2025). Menuet et al. (2025) highlight that respiratory modulation incorporates contributions from sympathetic activity and direct mechanical effects, specifically alterations in intrathoracic pressure that affect venous return and generate Traube-Hering waves in arterial pressure, in addition to the Bainbridge effect, where veno-atrial stretch contributes to the elevation of inspiratory heart rate (Menuet et al., 2025). Expanding on this perspective, Elstad and colleagues (2018) suggested that cardiorespiratory interactions are not limited to heart rate variability (RSA), but include distinct mechanisms such as cardioventilatory coupling (the temporal synchronization between heartbeat and the onset of inspiration) and the synchronization of stroke volume with respiration, suggesting that these integrated oscillations act to dampen systemic blood flow variability and optimize cardiac work efficiency (Elstad et al., 2018).

In summary, our results indicate that IMT not only increases resting vagal tone but also promotes an optimization of cardiorespiratory coupling. This improvement is manifested by changes in the RSA pattern and the modulation of HR responses to autonomic stress tests (Karim et al., 2023; Fisher et al., 2022). Consistent with the mechanisms of central crosstalk described by Menuet, these findings imply a strengthened neural interaction between respiratory and cardiovascular control centers. Furthermore, as proposed by Elstad et al. (2018), such synchronization, encompassing both neural drive and mechanical interactions, may enhance cardiac energetic efficiency and stabilize systemic blood flow (Elstad et al., 2018). Thus, these neural and mechanical adaptations suggest that IMT is a promising tool for the fine-tuning of cardiovascular regulation.

## Limitations

The current study has several limitations. First, adherence to the inspiratory muscle training protocol was self-reported, and the execution technique was directly observed only once a week during maximal inspiratory pressure testing and device load adjustments. Second, it should be noted that spectral analysis provides indirect indices of cardiac autonomic modulation based on cardiovascular and respiratory rhythmicity, rather than direct measures of vagal or sympathetic neural activity. Third, although changes in ventilatory timing (Ti/Ttot and expiratory duration) are compatible with central modulation of cardiorespiratory integration, direct markers of central respiratory motor output or respiratory drive were not assessed, precluding the distinction between central neural and peripheral mechanical contributions. Fourth, the imbalance in group allocation reduces statistical power and limits the strength of conclusions, particularly for secondary outcomes with marginal significance. Finally, the absence of female participants represents a limitation, as sex-related differences in cardiovascular autonomic modulation may exist.

## Conclusion

In conclusion, a 4-week IMT program increased inspiratory muscle strength, enhanced cardiac vagal modulation, and modified the cardiorespiratory interaction by shortening inspiratory time and lowering inspiratory heart rate, suggesting a reorganization of respiratory-cardiovascular timing. These results may contribute to advancing the understanding of the interaction between inspiratory muscle activity and the cardiorespiratory interactions.

## Data Availability

The raw data supporting the conclusions of this article will be made available by the authors, without undue reservation.
