# Supplementary material for: Effects of inspiratory muscle training on cardiorespiratory network physiology: evidence from cardiac autonomic modulation, respiratory sinus arrhythmia, and baroreflex sensitivity analysis
Source: Front Netw Physiol. 2026 Feb 17;6:1761610. doi: 10.3389/fnetp.2026.1761610 (PMC12953523; doi:10.3389/fnetp.2026.1761610)
Supplement: Supplementary file 1 [file Table1.docx]

Table S1 – Cardiovascular coupling and transfer entropy indexes at rest before and after IMT.

|  | PLA | | EXP | |  |  |
| --- | --- | --- | --- | --- | --- | --- |
|  | PRE_IMT | POST_IMT | PRE_IMT | POST_IMT | p-value |  |
| **Spontaneous breathing** | | | | | |  |
| RR Ro | 26.57±7.32 | 33.00±26.14 | 29.50±10.26 | 28.00±8.32 | 0.719 |  |
| RR Ce_Ro | 105.14±18.89 | 110.00±13.41 | 108.16± 18.26 | 106.41±17.24 | 0.899 |  |
| SAP Ro | 40.00±13.65 | 30.42±9.62 | 32.66±6.11 | 34.33±8.19 | 0.327 |  |
| SAP Ce_Ro | 91.00±18.10 | 109.85±14.89 | 99.83±13.17 | 97.08±17.57 | 0.479 |  |
| RR-SBP LF K^2^ | 71.00±30.33 | 61.57±27.72 | 73.75±18.27 | 87.25±13.15 | 0.312 |  |
| RR-SBP LF Ph | -56.14±327.80 | -234.85±186.40 | -334.08±174.00 | -254.66±275.64 | 0.296 |  |
| RR-SBP LF Gain | 79.71±46.84 | 81.71±45.29 | 103.51±20.57 | 117.02±33.40 | 0.524 |  |
| **Controlled breathing** | | | | | |  |
| RR Ro | 28.28±6.94 | 44.00±27.14 | 30.83±9.61 | 31.08±6.76 | 0.947 |  |
| RR Ce_Ro | 101.00±16.54 | 77.00±120.00 | 103.00±23.01 | 103.33±12.04 | 0.592 |  |
| SAP Ro | 38.71±10.60 | 99.28±16.99 | 34.16±8.08 | 39.66±9.71 | 0.825 |  |
| SAP Ce_Ro | 91.57±12.94 | 101.28±14.81 | 95.58±12.51 | 91.25±14.83 | 0.602 |  |
| RR-SBP LFHz | 85.85±42.83 | 112.42±32.43 | 107.75±21.26 | 111.08±19.57 | 0.778 |  |
| RR-SBP LF K^2^ | 58.28±39.91 | 75.85±13.70 | 73.91±20.24 | 85.00±13.76 | 0.290 |  |
| RR-SBP LF Ph | -72.71±475.29 | -216.85±228.77 | -280.25±240.73 | -184.66±225.63 | 0.205 |  |
| RR-SBP LF Gain | 92.22±21.25 | 68.40±22.82 | 100.85±30.35 | 118.11±44.64 | 0.063 |  |

PLA. placebo group; EXP. experimental group; IMT. inspiratory muscle training; Ro: Index of regularity; CE: conditional entropy; K^2^: Squared coherence; Ph: Phase direction; RR–SAP LF Gain: Baroreflex gain; RR: RR interval; SBP: systolic blood pressure.
